# G-Cimp Status Prediction Of Glioblastoma Samples Using mRNA Expression Data

**DOI:** 10.1371/journal.pone.0047839

**Published:** 2012-11-06

**Authors:** Mehmet Baysan, Serdar Bozdag, Margaret C. Cam, Svetlana Kotliarova, Susie Ahn, Jennifer Walling, Jonathan K. Killian, Holly Stevenson, Paul Meltzer, Howard A. Fine

**Affiliations:** 1 Neuro-Oncology Branch, National Cancer Institute, National Institutes of Health, Bethesda, Maryland, United States of America; 2 Department of Mathematics, Statistics, and Computer Science, Marquette University, Milwaukee, Wisconsin, United States of America; 3 Genetics Branch, National Cancer Institute, National Institutes of Health, Bethesda, Maryland, United States of America; 4 New York University Cancer Institute, New York University Langone Medical Center, New York, New York, United States of America; University of Michigan School of Medicine, United States of America

## Abstract

Glioblastoma Multiforme (GBM) is a tumor with high mortality and no known cure. The dramatic molecular and clinical heterogeneity seen in this tumor has led to attempts to define genetically similar subgroups of GBM with the hope of developing tumor specific therapies targeted to the unique biology within each of these subgroups. Recently, a subset of relatively favorable prognosis GBMs has been identified. These glioma CpG island methylator phenotype, or G-CIMP tumors, have distinct genomic copy number aberrations, DNA methylation patterns, and (mRNA) expression profiles compared to other GBMs. While the standard method for identifying G-CIMP tumors is based on genome-wide DNA methylation data, such data is often not available compared to the more widely available gene expression data. In this study, we have developed and evaluated a method to predict the G-CIMP status of GBM samples based solely on gene expression data.

## Introduction

Glioblastoma Multiforme (GBM) is a deadly brain tumor with few effective therapies. Identification of the underlying pathogenic mechanisms involved in the initiation and progression of this tumor is critical for developing more effective treatments. Recent studies have demonstrated the profound genetic and molecular heterogeneity of GBMs. This molecular heterogeneity complicates the identification of the core elements within the cellular signaling network of any given GBM thereby limiting out ability to offer targeted therapies for a specific tumor.

Recent developments in genomic technology (microarray, next generation sequencing etc.) have enabled a large number of GBMs to be genetically characterized at unprecedented levels of detail. Although such studies have revealed the heterogeneity between GBMs, they have also allowed the identification of subgroups of tumors that are more closely related than others. A number of recent studies using supervised and unsupervised analyses have been published stratifying GBMs into similar subgroups based on mRNA expression profiles [1], [2], [3], [4].

Our understanding of the epigenome has improved substantially in the last decade. In particular, epigenetic biomarkers such as DNA methylation and their effects on tumor biology have been analyzed in a number of GBM studies [5]. With the recent development of high-resolution microarray platforms, it is now possible to measure the level of methylation across an entire genome. Two such platforms are the Illumina Infinium 27 k and 450 k platforms. These platforms report the methylation ratio based on methylated and unmethylated probe intensities. The main difference between these platforms is the number of methylation sites included. These platforms have been shown to have high concordance with each other and with bisulfite-sequencing [6], [7], [8].

The ability to acquire whole genome-wide DNA methylation data has opened up the possibility for its use as an alternative classification methodology, as demonstrated recently in GBM [9]. In this study, the authors describe two clearly separated clusters, defined as G-CIMP positive and G-CIMP negative, with the G-CIMP positive group comprising less than 10% of all GBM samples. As a group, patients with G-CIMP positive GBMs tended to be younger, have fewer genomic alterations and show better survival than G-CIMP negative patients. A predominant genetic feature of G-CIMP positive GBMs is the frequent mutation of IDH1, which is rarely found in G-CIMP negative GBMs. Recently, IDH1 mutations have been associated with the altered methylation profiles in G-CIMP positive gliomas [10].

Secondary to the fact that the G-CIMP status of GBMs identifies a group of patients with genetically distinct tumors with very different clinical outcomes, there is an increasing need to identify such tumors clinically. Unfortunately few clinical or even research laboratories generate whole genome methylation data on tumor specimens making the identification of G-CIMP tumors impossible. By contrast, an increasingly larger number of laboratories are generating mRNA expression data from their clinical tumor specimens. In this study, we demonstrate how a computational algorithm we have devised allows one to identify the G-CIMP phenotype of any given GBM at nearly 100% accuracy using only mRNA expression data.

## Materials and Methods

### Samples

We used public TCGA (http://cancergenome.nih.gov/) and REMBRANDT (http://caintegrator-info.nci.nih.gov/rembrandt) data repositories as our primary source of samples. For methylation-based validation, we used twenty-one GBMs and one non-tumor brain sample. Twenty-one GBM samples were obtained from the prospective NCI-sponsored Glioma Molecular Diagnostic Initiative (GMDI), which were provided as snap frozen sections. Pathological diagnosis of these samples was determined by the local institutional neuropathologist and centrally reviewed by two NIH neuropathologists who were blinded to the original diagnosis. Only tumors that met the criteria of having a consensus pathological diagnosis from the NIH neuropathologists were utilized for our analyses. The one non-tumor sample was obtained from a medically indicated therapeutic temporal lobe resection from an NIH patient with refractory epilepsy.

### Methylation Experiment

We isolated DNA from cell pellets and fresh frozen tumor tissue using the QIAmp DNA micro kit (Qiagen). One microgram of the DNA was bisulfite converted and processed on Human Methylation450 BeadChips (Illumina) using the Infinium HD Methylation Assay as described previously [11]. We interrogated 485,000 individual CpG sites per sample at single-nucleotide resolution. Image data were extracted and analyzed using the GenomeStudio v2010.3 methylation module (Illumina).

### Data Sets

#### TCGA methylation data set

We used level-2 methylation data from Illumina Infinium 27 k and 450 k platforms. For the 27 k methylation data we filtered 23,487/27,578 sites with no missing values on non-sex chromosomes. Similarly there were 486,412 methylation sites with no missing values retained in 450 k methylation data. We found 22,270 of these methylation sites to be shared between 27 k and 450 k platforms.

Methylation data sets from both platforms based on common methylation sites were then filtered to 1509 sites which had a standard deviation of 0.2 or greater as in [9]. There were 368 samples and 1509 methylation sites in our final TCGA methylation data set.

#### TCGA expression data set

We imported 413 Affymetrix U133A samples (raw data) using RMA from the TCGA portal (RMA background correction, quantile normalization, median polish). There were 403 GBM and 10 normal brain samples in this data set. We performed unsupervised clustering based on 1670 probe sets (std. dev.>1) to check if any GBM samples cluster with normal samples due to possible normal tissue contamination. Three samples clustered with normal brain samples and were excluded from data set (Figure S1).

#### NOB expression data set

We used gene expression data for 201 GBM and 31 normal brain samples in the NOB database. Most of these samples have been publicized in REMBRANDT public repository previously [12]. These samples were generated using Affymetrix U133 plus 2.0-microarray platform using the 1-cycle protocol. We normalized the data using RMA (RMA background correction, quantile normalization, median polish). We performed unsupervised clustering on 2178/54,675 high variation (standard deviation>1) probe sets. We detected 27 GBM samples, which clustered with normal samples. These samples were excluded due to likely normal brain contamination (Figure S2).

#### NOB methylation data set

Raw methylation data imported using GenomeStudio Software (include ref version). Methylation sites, which have detection p-value less than 0.05 for all samples, have been retained.

### Batch Removal

We used a mixed model ANOVA based algorithm by Partek 6.6beta (Partek™ software (Partek Inc., St. Charles, MI)) to remove the batch effects between TCGA and NOB gene expression data sets. To measure the efficiency of batch effect removal, we used gene expression data for normal brain samples. We assumed that normal brain samples in both data sets would cluster together in a combined data set if the batch effect removal were successful.

There were two variables within the ANOVA model that were used for batch removal. These variables are sample origin (TCGA or NOB) and tissue origin (GBM or normal). We first showed that normal brain samples from two different data sources cluster together after batch effect removal; from this we concluded that the batch effect removal was successful. We then excluded non-tumor samples from both data sets and applied batch effect removal on sample origin to obtain final data set.

### Hierarchical Clustering (HC) and Principle Component Analysis (PCA)

We used Partek Software (6.6 beta,Partek Inc., St. Charles, MI) to perform HC and PCA. Average linkage and Euclidean distance were used for all HCs. Expression data was standardized by sample columns prior to performing the HC, although this was not done for the methylation data. PCAs were performed using correlation dispersion matrix and normalized eigenvector scaling.

### G-CIMP Prediction Based on Expression Data

We generated five prediction models, which differ only by the use of the top number of differentially expressed probe sets (10, 25, 50, 100 and 200). All models used nearest neighbor algorithm, which classifies unknown samples based on the closest known sample. Euclidean distance was used as the distance measure. We used the Partek implementation of this algorithm. Probe sets included in these five models are provided as supplementary files (Table S1, S2, S3, S4, S5).

## Results

### Unsupervised Clustering of TCGA Samples Based on Methylation Data

We obtained TCGA methylation data for 368 GBM samples and performed unsupervised clustering to identify G-CIMP status of these samples as described in [9] (see Materials and Methods). We applied partition clustering with different number of clusters (2–5) and obtained the best performance (Davies-Boudin Score) by clustering into two groups (Figure S3).

**Figure 1:**
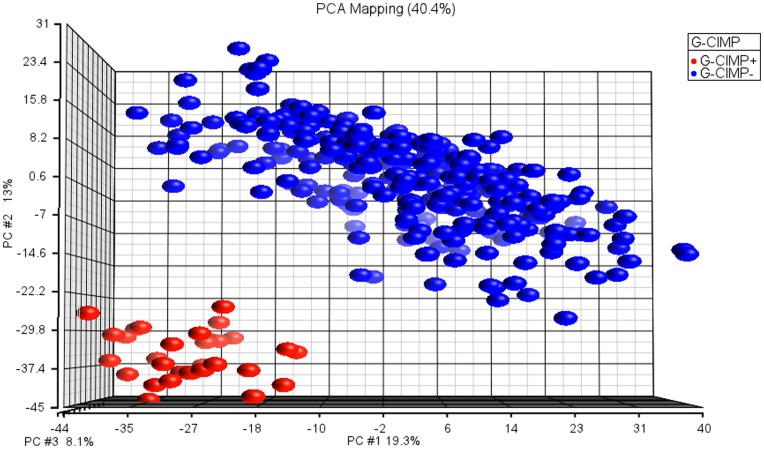
PCA plot for TCGA samples based on methylation data (Blue: G-CIMP positive, red: G-CIMP negative samples). 1509 methylation sites (std. dev. >0.2) have been shown.

We labeled the larger cluster (338 samples) as G-CIMP negative and the smaller cluster (30 samples) as G-CIMP positive (Figure 1). TCGA recently released a Data Freeze package, which includes the G-CIMP calls (TCGA Analysis Working Group Data Release Package, 9/3/2011, https://wiki.nci.nih.gov/display/TCGAM/DatasetsGBM). 365 of the 368 samples we analyzed had an existing G-CIMP classification in the TCGA package. Out of the 365 samples, only four samples were found to have contradicting labels, i.e. they are labeled as G-CIMP positive in TCGA and as G-CIMP negative in our list (Table 1). According to our unsupervised analysis, these samples are clearly clustered with G-CIMP negative samples (Figure S4). Thus, we decided to keep these samples in our subsequent analyses.

**Table 1:** Comparison of TCGA G-CIMP labels and our G-CIMP calls.

	TCGA G-CIMP positive	TCGA G-CIMP negative	TCGA UNKNOWN
Predicted G-CIMP positive	30	0	0
Predcited G-CIMP negative	4	331	3

### Prediction of G-CIMP Calls of TCGA Samples from Expression Data

There are many samples in the TCGA repository where mRNA expression data is available but more comprehensive genome-wide methylation data (27 k or 450 k) is not available. We hypothesized that the G-CIMP calls of these GBM samples could be predicted based on gene expression data. To test this hypothesis, we used the gene expression data set for samples with available G-CIMP calls (based on methylation) as the training data. After training five prediction models, we predicted the G-CIMP status of the subset of TCGA samples that had available expression data but not methylation data. The difference between these five models was the use of either the top 10, 25, 50, 100 or 200 differentially expressed probe sets between the G-CIMP positive and negative groups, respectively.

We obtained expression profiles of 403 GBMs and ten normal brain samples using the Affymetrix U133A platform from the TCGA data repository. We performed a principal component analysis and observed that three of the GBM samples cluster with normal brain samples (Figure S5). These samples were eliminated in our analysis due to possible contamination with normal brain tissue.

The G-CIMP status of 218 out of 400 GBM samples was available from methylation-based classifications (last section). Before predicting the G-CIMP status of the remaining samples, we used samples with available G-CIMP data to measure the prediction performance on expression data by applying a method called *two-fold cross validation*. Briefly, we divided the sample set with known G-CIMP status into two groups and labeled one of these groups as the training set and the other group as the test set. We developed a prediction model based on the expression data of the training set and applied this model on the test set (see Materials and Methods). Then, we compared the real labels with predictions. We repeated this operation by changing the training and test sets. We obtained 100% prediction accuracy rate in the cross-validation (Table S6).

After validating the performance of our prediction algorithm, we used it to predict the G-CIMP status of the subset of TCGA samples that did not have methylation data. Following our prediction, there were 30 G-CIMP positive and 370 G-CIMP negative samples in the TCGA expression data set.

### Prediction of G-CIMP Calls Using an Alternate Data Set (REMBRANDT)

In order to evaluate the robustness of our prediction algorithm, we obtained gene expression profiles of 174 GBM samples in the NOB/REMBRANDT database after *contamination filtering* (see Materials and Methods). We combined the NOB expression data set and the TCGA expression data set using 22,277 common probe sets. We observed a significant batch effect between TCGA and NOB gene expression data sets (Figure S6, Figure S7). This result is expected because the TCGA data set is based on the Affymetrix U133A platform using the IVT labeling protocol, while the NOB data set is based on the Affymetrix U133 Plus 2.0 platform and 1-cycle target labeling protocol (www.affymetrix.com). We removed this batch effect by using an ANOVA based batch effect removal algorithm (see Materials and Methods).

**Figure 2:**
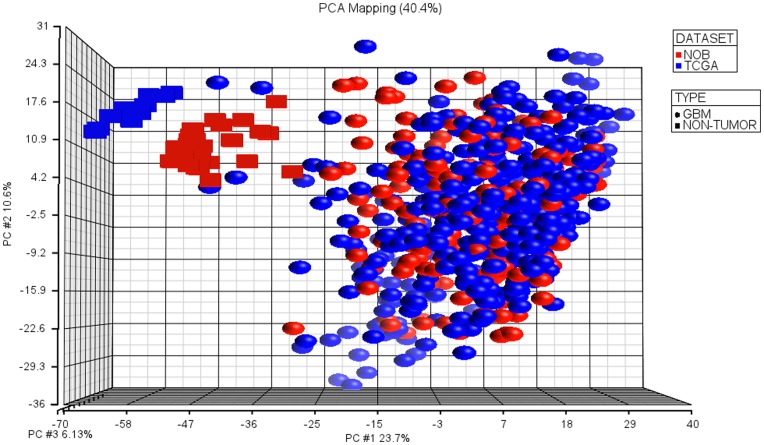
PCA plot of TCGA and NOB samples after batch effect removal. As expected, normal brain samples are clustered together, and TCGA and NOB samples are not separated. We used high variation 1482 probe sets (std. dev. >1) in combined data set.

After batch effect removal, the separation between TCGA and NOB samples diminished and normal brain samples from both data sets clustered together which shows the adequacy of the batch effect removal (Figure S6, Figure S7 versus Figure S8, Figure 2).

**Figure 3:**
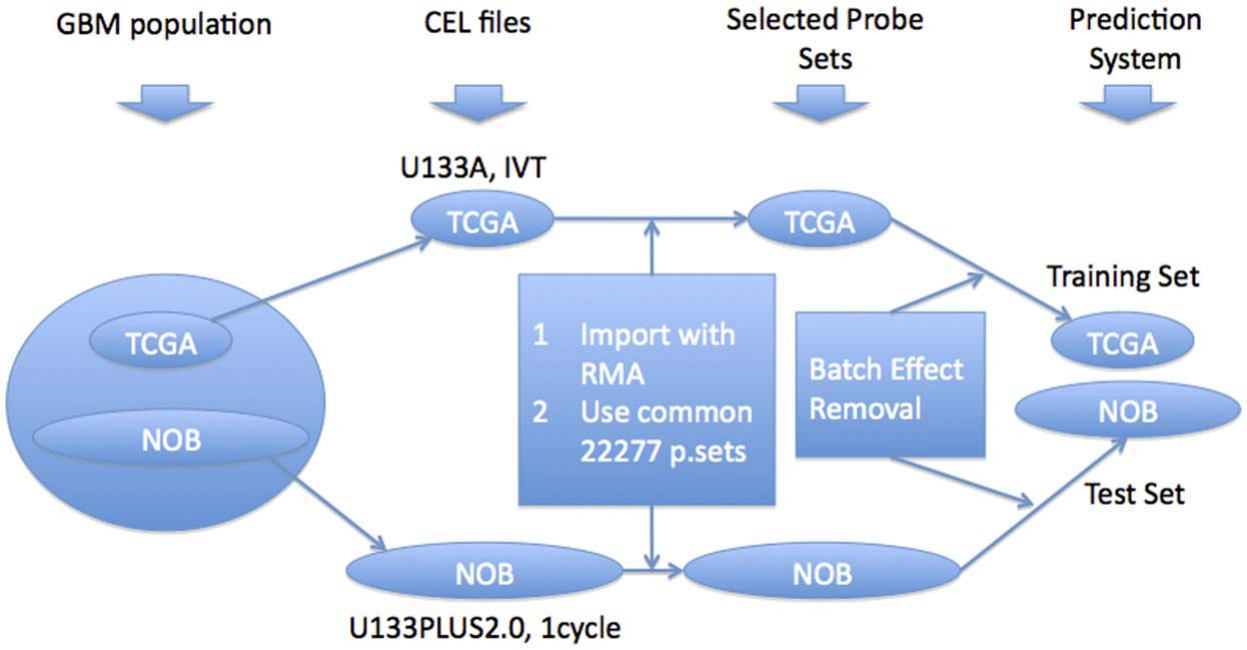
Data processing flowchart for predicting G-CIMP calls of NOB samples from TCGA gene expression data.

We constructed our training and test sets as follows (Figure 3): we imported and normalized CEL files using RMA for each data set independently (400 TCGA samples and 174 NOB samples) and selected only the common 22,227 probe sets. We repeated the batch effect removal step between TCGA and NOB data sets without including the normal brain samples. We assumed that the batch effect removal would be more successful without normal samples as the data sets become more homogeneous without normal samples. We assigned the TCGA data as the training set and the NOB data as the test set.

We then built five different prediction models based on the training set (i.e., TCGA dataset) using the top 10, 25, 50, 100, and 200 differentially expressed probe sets between G-CIMP positive and negative samples. We repeated the two-fold cross-validation within the TCGA expression data set. The two-fold cross-validation in the previous section would not suffice since (i) earlier cross-validation was within only 218 GBM samples, and (ii) the gene expression data set had been manipulated substantially with the batch effect removal. The cross-validation results achieved 99.75% accuracy for all models.

We applied all five models on NOB samples to predict their G-CIMP status. Five models agreed on 10 samples as G-CIMP positive and 159 samples as G-CIMP negative. There was no consensus for the remaining five samples, which were labeled as Non-Consistent (NC).

### Clinical Validation

**Figure 4:**
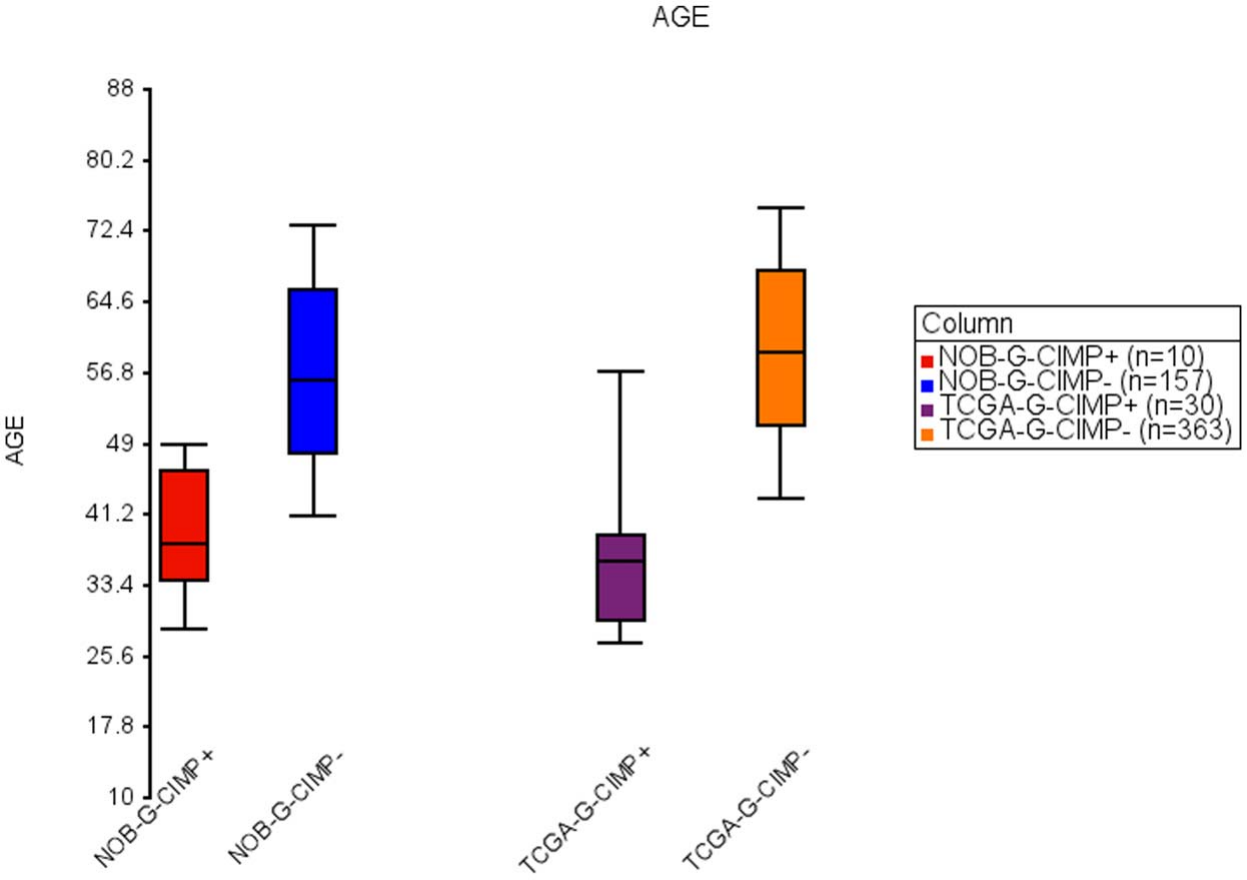
Box-Whisker plot of age of NOB and TCGA G-CIMP positive and G-CIMP negative samples. Limits of box and whiskers plot are 10%, 25%, 50%, 75% and 90%.

It has been previously demonstrated that there are significant age and survival differences between patients with G-CIMP positive and G-CIMP negative GBMs [9]. In our analysis, the median age for TCGA G-CIMP positive GBMs was 36 compared to the median age for TCGA G-CIMP negative GBMs that was 59 (t-test p-value <1.628e-15). Similarly, the median age for the predicted NOB G-CIMP positive GBMs was 38 compared to 56 years for the median age of G-CIMP negative GBMs (t-test p-value <2.1968e-05) (Figure 4).

**Figure 5:**
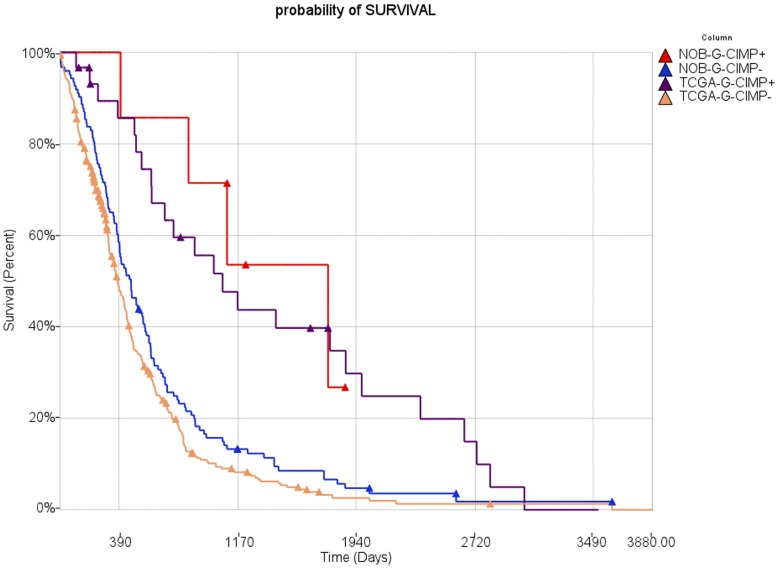
Kaplan-Meier survival plot for NOB G-CIMP+ (n = 7), NOB G-CIMP− (n = 123), TCGA G-CIMP+ (n = 30) and TCGA G-CIMP− (n = 361) samples.

The calculated median survival for TCGA G-CIMP positive GBMs in our analysis was 840.5 days whereas the median survival for TCGA G-CIMP negative GBMs was 327 days (Wilcoxon-Gehan p-value <1.10279e-05). Consistent with these data, the calculated median survival for the predicted G-CIMP positive NOB samples was 1121 days whereas median survival for predicted G-CIMP negative NOB samples was 470 days (Wilcoxon-Gehan p-value <0.0103) (Figure 5). These results show that, as expected, predicted NOB G-CIMP positive patients were younger and survived significantly longer than predicted NOB G-CIMP negative patients. Moreover, the median age and survival of NOB G-CIMP positive/negative and TCGA G-CIMP positive/negative samples are very similar, respectively.

### Validation of G-CIMP Prediction by Methylation Assay

We used the Illumina Human Methylation 450 K platform to generate methylation data on a subset of the NOB samples to validate our predictions. We selected nine G-CIMP positive samples, seven G-CIMP negative samples, all five NC samples, and one normal brain sample for the methylation assays (see Materials and Methods).

We combined our methylation data set with the TCGA methylation data set methylation data from four normal brain samples provided by USC Epigenome Center (http://epigenome.usc.edu). In this combined data set, 1438 out of 1509 methylation sites passed through our detection filter.

**Figure 6:**
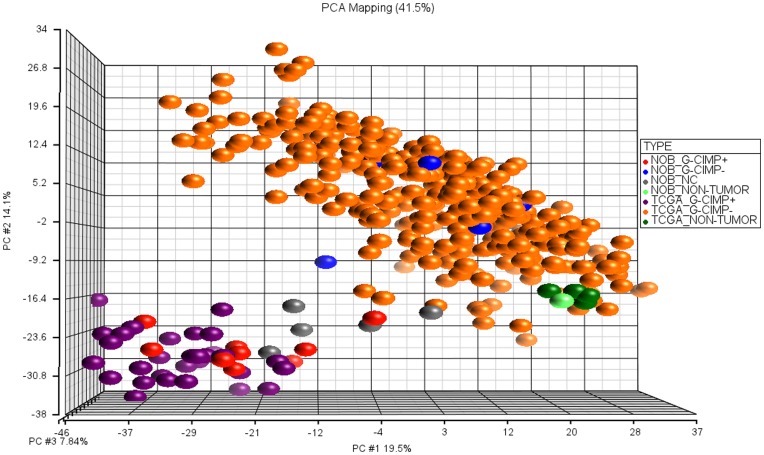
PCA plot of TCGA and NOB samples in combined methylation data set. This data set includes 394 samples and 1438 methylation sites.

First, we checked if there was a batch effect since the data sets came from different groups (i.e., NOB vs. TCGA). We observed that normal samples clustered tightly in the combined data set suggesting that the batch effect between data sets was minimal (Figure 6). After demonstrating the lack of a significant batch effect in the combined data set, we compared expression-based G-CIMP labels to methylation-based G-CIMP labels. We observed that in general, predicted G-CIMP positive samples clustered with TCGA G-CIMP positive samples and predicted G-CIMP negative samples clustered with TCGA G-CIMP negative samples based on methylation data (Figure 6). We then classified our methylation samples into G-CIMP subtypes using the TCGA samples as the training set, using nearest neighbor algorithm, Euclidean distance and all 1438 methylation sites. We found 100% accuracy in a two-fold cross validation of TCGA GBM samples. We found only one mis-prediction in our samples; one G-CIMP negative sample (on methylation data) had been predicted as G-CIMP positive (on expression data).

**Figure 7:**
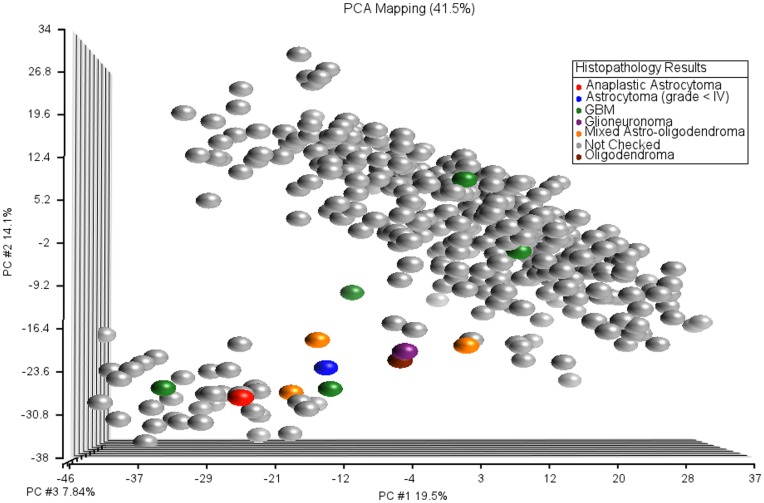
PCA plot of TCGA and NOB samples in combined methylation data set. Samples are labeled by histopathology check results.

We noticed that the mis-predicted sample and a majority of the NC samples fell between the two G-CIMP methylation clusters (Figure 6). In the original TCGA methylation data set, the two G-CIMP clusters were clearly separated with no samples showing an intermediate methylation profile. This made us suspicious about these samples that fell between the G-CIMP positive and G-CIMP negative clusters. We reevaluated twelve NOB samples based on clinical reports (Figure 7). Six of these twelve samples were ambiguous (5 NC and one mis-predicted) and remaining six were non–ambiguous. Five of the six non-ambiguous samples were GBMs and remaining one was Anaplastic Astrocytoma. On the other hand, all six ambiguous samples were discovered not to be GBMs. Thus, the difficulty in classifying some of the samples was determined to be related to poor sample labeling. Thus, our prediction algorithm was found to have 100% accuracy for correctly labeled GBM samples.

Of interest, we observed many NCs when we tried to classify non-tumor samples into G-CIMP subtypes. Thus, it appears that our classification system has difficulty in characterizing non-GBM pathology. This probably reflects the fact that G-CIMP biology is unique to GBMs and cannot be applied to other tumor types and/or normal tissue.

### Methylation Status and Biological Interpretation of the Genes in the Prediction Models

In order to evaluate the methylation status and perform functional enrichment analysis for the genes in our prediction models we first analyzed the genes in prediction models in terms of methylation difference between G-CIMP positive and G-CIMP negative subtypes. Next, we analyzed the genes in the prediction models in terms of methylation-expression correlation. Finally, we uploaded the genes in our prediction models to Ingenuity Pathway Analysis (IPA) to inquire whether these genes show enrichment for specific biological processes.

Our prediction models used probe sets, which represent mRNA expression. By contrast, methylation data is based on the methylation ratio of a site on a given gene. We combined these two data types at the gene level. We converted the probe sets to gene names and generated six categories based on the existence of genes in our prediction models as follows. If a gene is included in our first prediction model with ten probe sets, it is assigned in the first category, which is denoted by *top10*. If it is not in the first prediction model but in the second prediction model, which has twenty-five probe sets, it is in the second category, which is denoted by *top11*–*25*. Similarly, we created three more categories denoted by *top26–50, top51–100* and *top101–200*. If a gene is not included in any of the five prediction models we used, it is assigned to the last category, which is denoted by not-in-lists.

After defining the categories, we compared the methylation status for the genes in each category. First, we calculated the median methylation for each methylation site, for G-CIMP positive group and G-CIMP negative group. Accordingly, we used the Beta-values in TCGA methylation data set we had created (Beta values roughly represent the ratio between methylated and unmethylated probe intensities.). Then, we measured the absolute median methylation difference by subtracting these numbers and taking the absolute value. Next, we compared the genes in six categories in terms of absolute median methylation difference. We observed that genes we used in prediction models indicated higher methylation difference compared to genes that are not included (Figure S9).

We next compared the methylation-expression correlations among the six categories by computing the methylation-expression Pearson correlation for each methylation site using Beta-values and TCGA Level-3 expression data (Agilent Platform). When we compare the methylation-expression correlations for the genes in the six categories, we observed a strong negative correlation for the genes in our prediction models (Figure S10). These results demonstrate that selected genes have clear methylation differences and these differences are reflected on gene expression.

We next used IPA to assess the functional enrichment for the genes in our prediction models. We identified the genes that are included in the prediction model with 200 probe sets and uploaded these genes to IPA along with the gene names and expression fold changes between G-CIMP positive and G-CIMP negative samples using TCGA expression data set. IPA reported only one significant function in its functional enrichment module, that being an increase in ‘proliferation of cells’ associated genes in G-CIMP negative GBMs. This result is consistent with the poor survival rate of G-CIMP negative GBM patients. In the transcription factor activity module, IPA identified KDM5B (lysine (K)-specific demethylase 5B) as the most significantly altered transcription factor. According to IPA report, KDM5B is activated in G-CIMP positive samples. It is interesting to observe a demethylase with increased activity in G-CIMP subtype considering the altered methylation profile in this subtype [10].

### Importance of Proper Subtyping

Although the literature is replete with studies finding correlations between patient clinical outcome (e.g. survival) and specific genotypes (e.g. gene expression, chromosomal number variations), the majority of such studies have treated GBM as a homogeneous disease. This approach might have led to false interpretations if different genomic subtypes of GBM have different clinical outcomes. For instance, it is possible that some of these “survival-specific” genes in GBM could be enriched for in G-CIMP tumors. Similarly, a disproportionate number of patients with G-CIMP positive or negative tumors could influence the outcome of a clinical trial and be falsely interpreted as a therapeutic effect.

To measure the effect of G-CIMP status on survival, we ran a Cox-regression for all genes with and without the G-CIMP samples. We found 200 and 3 survival-correlated probe sets in TCGA (n = 391) and the NOB data (n = 134), respectively with a FDR<0.05 (Benjamini-Hochberg step-up multiple test correction [13]). We could not find any survival-correlated probe sets in both data sets after removing G-CIMP positive samples. This suggests that all survival-related genes we identified were related to their disproportional representation in the G-CIMP tumors and the overall improved survival of this group of patients.

Using less stringent criteria (p<0.05) to measure the G-CIMP effect on survival, we found by a Cox-regression analysis 3258 probe sets related to survival for all samples with survival information (n = 391) in the TCGA data set. By contrast, there were only 986 probe sets related to survival when G-CIMP positive samples were excluded (n = 361, 489 probe sets are shared between two lists). Similarly, using NOB data we found 2377 probe sets related to survival for all samples (n = 134) but only 1142 probe sets for G-CIMP negative samples (n = 123, 937 probe sets are shared between two lists).

These results show that more than 84% of the survival-related probes sets in the initial TCGA dataset are changed when G-CIMP positive samples are removed (60% in the NOB dataset). Moreover the number of survival-related probe sets identified is reduced by 70% after removing G-CIMP samples in the TCGA data set and by 52% in the NOB data set. Although reducing the sample size will lower the statistical power to identify survival related probe sets, the less than 8% reduction in sample size is unlikely to be responsible for the large decrease in survival related probe sets when we look only at G-CIMP negative tumors. In order to roughly measure the affect of sample size on the number of survival related probe sets, we randomly deleted ∼10% (36/361) of G-CIMP negative samples and repeated the analyses. In the new data set, 39% of probe sets have been changed (381 out of 986 probe sets) and the number of probe sets has been reduced by only 10% (986 to 881 probe sets).

## Discussion

In this study, we have established a method to classify GBM samples into G-CIMP subtypes based on gene expression data. As expected, we have observed a high concordance between the TCGA G-CIMP groups and predicted NOB G-CIMP groups in terms of age and survival. We have also validated the methylation differences between NOB G-CIMP positive and negative samples by performing in vitro methylation assays. These results demonstrate that the G-CIMP subtypes are distinct and can be reproducibly validated on a totally independent data set. An alternative approach to identify G-CIMP positive GBMs would be to check for IDH1 mutations. However, a small but significant subset of G-CIMP positive GBMs (5/23 in [9]) do not carry this mutation thus reducing the accuracy of such an approach.

We used the TCGA Affymetrix U133A data set as training data because the NOB expression data was based on the Affymetrix U133PLUS2.0 platform which uses a similar technology to U133A. TCGA has expression data on multiple platforms such as the Agilent 244 K G4502A and Affymetrix Human Exon 1.0 platforms. Investigators interested in adopting our method for G-CIMP classification and have expression data from any of the array platforms used by TCGA can simply apply our methods by using the corresponding TCGA data set as training data.

Since multidimensional clustering algorithms are able to produce subclasses even on random data, it is important to validate clusters on an external dataset. There are well-known differences between microarray data produced in different settings (lab, platform, protocol) [14]. These batch effects can limit the reproducibility of observed subtypes in external data sets unless the differences across the subtypes are large enough. This limitation holds for other biological results besides subtypes. Although independent validation takes time, without such validation one is likely to report a number of false positives. Accordingly, recent reports [15], [16] have shown limited reproducibility among multiple microarray studies. A recent study explored the possible causes for the lack of consensus in attempting to derive expression-based subtypes of GBM [17].

Batch effects can be removed via two general mechanisms. One of these involves running identical specimens (also called batch controls) on both data sets. Both data sets could then be scaled to make these identical specimens equivalent on the combined data set. Another method involves combining data sets based on a representation assumption. If two data sets are *large enough, then sampling the same population* allows one to assume they *represent* the same entity. In this case, we can apply batch effect removal algorithms that scale the data sets and make them statistically comparable. However, if data sets are *not large enough* or they are not derived from *identical populations,* then the representation assumption fails and the results will be flawed. In this instance, a GBM data set may not be combined with a general brain tumor data set although one population is the subset of the other. Similarly we cannot combine two GBM data sets that contain tumors from patients with disproportionate distributions of age or sex unless we show that there is no molecular difference between these different clinical groups.

In this vein, the establishment of gene classifiers is also based on the assumption that the original data on which the classifiers were developed and the new data on which the classifiers are to be used, represent the same entity. If data sets are not large enough or they represent different populations this operation would be flawed and might lead to false interpretations. For example, if a gene signature, which separates different subsets of GBM is applied to a general brain tumor data set, results are likely to be unreliable. We saw this in our analysis where our G-CIMP classifiers gave us indeterminate results when we used them to attempt to classify non-GBM gliomas and normal brain tissue.

One issue that would potentially negatively affect the accuracy of our analysis concerns the origin of the tumor samples. The TCGA samples are generally selected from untreated primary GBMs while the NOB tumor samples are mostly previously treated and recurrent GBMs. Interestingly, our analyses demonstrate that this difference did not affect our ability to identify the G-CIMP subtypes. In fact, the similarity between TCGA and NOB data in terms of age and survival profiles of the derived G-CIMP positive and G-CIMP negative groups were excellent.

Unlike the situation with gene expression data, we did not observe any separation or batch effect between the two un-normalized methylation data sets but rather found that the non-tumor samples from both data sets clustered tightly. This positive outcome suggests that Infinium methylation data sets from different labs are comparable without data manipulation. This is a major advantage that will allow increasing statistical power for future analyses by creating larger data sets by combining multiple smaller data sets.

Identifying and assigning patient-specific tumors to stable genetic sub-types in heterogeneous diseases like GBM will be an important step towards personalized medicine. Alternative therapeutic regimes can be developed for different subtypes, and should target subtype specific vulnerabilities. Establishing the stability and reproducibility (universality) of these subtypes is a critical step in achieving this goal. Our study demonstrates that G-CIMP methylation subtypes are stable enough to be independently validated through gene expression array data despite strong batch effects. Moreover we demonstrate that the prognostic significance of the G-CIMP subtype is very consistent, even when evaluated across two totally independent large cohorts of patients. This result suggests that identification of the G-CIMP will have significant clinical relevance for clinical trial design stratification, patient prognosis and potentially treatment in the future.

## Supporting Information

Figure S1 3 TCGA GBM samples are clustered with normal brain samples and filtered out from TCGA expression data set.
(DOC)

Figure S2 27 NOB GBM samples are clustered with normal brain samples and filtered out from NOB expression data set.
(DOC)

Figure S3 Davies-Boudin clustering performance score for clustering on TCGA methylation data for various number of clusters. Clustering separation is better when this score is lower.
(DOC)

Figure S4 1438 methylation sites. Four samples show disagreement between TCGA and our G-CIMP calls.
(DOC)

Figure S5 PCA plot of TCGA GBM and normal brain samples based on gene expression data.
(DOC)

Figure S6 (A,B) PCA plots of GBM and normal brain samples in TCGA and NOB gene expression data sets before batch effect removal. 506 probe sets with largest variation are shown (C) Sample histogram of GBM and normal brain samples in TCGA and NOB gene expression data sets. We have used common 22,277 probe sets between Affymetrix U133 Plus 2.0 and Affymetrix U133A platforms.
(DOC)

Figure S7 Hierarchical clustering of GBM and normal brain samples in TCGA and NOB gene expression data sets before batch effect removal. We used high variation 506 probe sets of combined data set for hierarchical clustering.
(DOC)

Figure S8 (A,B) PCA plots of GBM and normal brain samples in TCGA and NOB gene expression data sets after batch effect removal. 742 probe sets with largest variation are shown (C) Sample histogram of GBM and normal brain samples in TCGA and NOB gene expression data sets. We have used common 22,277 probe sets between Affymetrix U133 Plus 2.0 and Affymetrix U133A platforms.
(DOC)

Figure S9 Methylation sites in different categories are represented with respect to absolute median methylation difference between G-CIMP positive and G-CIMP negative subtypes.
(DOC)

Figure S10 Methylation sites in different categories are represented with respect to methylation-expression correlations (Pearson).
(DOC)

Table S1 Prediction models with 10 probe sets.
(DOCX)

Table S2 Prediction models with 25 probe sets.
(DOCX)

Table S3 Prediction models with 50 probe sets.
(DOCX)

Table S4 Prediction models with 100 probe sets.
(DOCX)

Table S5 Prediction models with 200 probe sets.
(DOCX)

Table S6 Prediction accuracy of the G-CIMP prediction method based on two-fold cross validation on the samples with known G-CIMP calls.
(DOCX)

## References

[1] Li A, Walling J, Ahn S, Kotliarov Y, Su Q, et al (2009) Unsupervised analysis of transcriptomic profiles reveals six glioma subtypes. Cancer research 69: 2091. 19244127 10.1158/0008-5472.CAN-08-2100 PMC2845963

[2] Phillips HS, Kharbanda S, Chen R, Forrest WF, Soriano RH, et al (2006) Molecular subclasses of high-grade glioma predict prognosis, delineate a pattern of disease progression, and resemble stages in neurogenesis. Cancer cell 9: 157–173. 16530701 10.1016/j.ccr.2006.02.019

[3] Verhaak RGW, Hoadley KA, Purdom E, Wang V, Qi Y, et al (2010) Integrated genomic analysis identifies clinically relevant subtypes of glioblastoma characterized by abnormalities in PDGFRA, IDH1, EGFR, and NF1. Cancer cell 17: 98–110. 20129251 10.1016/j.ccr.2009.12.020 PMC2818769

[4] Huse JT, Phillips HS, Brennan CW (2011) Molecular subclassification of diffuse gliomas: seeing order in the chaos. Glia. 10.1002/glia.21165 21446051

[5] Malzkorn B, Wolter M, Riemenschneider MJ, Reifenberger G (2011) Unraveling the Glioma Epigenome' ÄîFrom Molecular Mechanisms to Novel Biomarkers and Therapeutic Targets. Brain Pathology 21: 619–632. 21939466 10.1111/j.1750-3639.2011.00536.x PMC8094062

[6] Bibikova M, Barnes B, Tsan C, Ho V, Klotzle B, et al.. (2011) High density DNA methylation array with single CpG site resolution. Genomics. 10.1016/j.ygeno.2011.07.007 21839163

[7] Dedeurwaerder S, Defrance M, Calonne E, Denis H, Sotiriou C, et al (2011) Evaluation of the Infinium Methylation 450 K technology. Epigenomics 3: 771–784. 22126295 10.2217/epi.11.105

[8] Sandoval J, Heyn HA, Moran S, Serra-Musach J, Pujana MA, et al (2011) Validation of a DNA methylation microarray for 450,000 CpG sites in the human genome. Epigenetics official journal of the DNA Methylation Society 6: 692–702. 10.4161/epi.6.6.16196 21593595

[9] Noushmehr H, Weisenberger DJ, Diefes K, Phillips HS, Pujara K, et al (2010) Identification of a CpG Island Methylator Phenotype that Defines a Distinct Subgroup of Glioma. Cancer cell 17: 510–522. 20399149 10.1016/j.ccr.2010.03.017 PMC2872684

[10] Turcan S, Rohle D, Goenka A, Walsh LA, Fang F, et al.. (2012) IDH1 mutation is sufficient to establish the glioma hypermethylator phenotype. Nature. 10.1038/nature10866 PMC3351699 22343889

[11] Rechache NS, Wang Y, Stevenson HS, Killian JK, Edelman DC, et al.. (2012) DNA Methylation Profiling Identifies Global Methylation Differences and Markers of Adrenocortical Tumors. Journal of Clinical Endocrinology & Metabolism. 10.1210/jc.2011-3298 PMC3387424 22472567

[12] Madhavan S, Zenklusen JC, Kotliarov Y, Sahni H, Fine HA, et al (2009) Rembrandt: helping personalized medicine become a reality through integrative translational research. Molecular Cancer Research 7: 157. 19208739 10.1158/1541-7786.MCR-08-0435 PMC2645472

[13] Benjamini Y, Hochberg Y (1995) Controlling the false discovery rate: a practical and powerful approach to multiple testing. Journal of the Royal Statistical Society Series B (Methodological): 289–300.

[14] Luo J, Schumacher M, Scherer A, Sanoudou D, Megherbi D, et al (2010) A comparison of batch effect removal methods for enhancement of prediction performance using MAQC-II microarray gene expression data. The pharmacogenomics journal 10: 278–291. 20676067 10.1038/tpj.2010.57 PMC2920074

[15] Ioannidis JPA, Khoury MJ (2011) Improving Validation Practices in, ÄúOmics' Äù Research. Science 334: 1230–1232. 22144616 10.1126/science.1211811 PMC5624327

[16] Peng RD (2011) Reproducible Research in Computational Science. Science 334: 1226–1227. 22144613 10.1126/science.1213847 PMC3383002

[17] Marko NF, Quackenbush J, Weil RJ (2011) Why Is There a Lack of Consensus on Molecular Subgroups of Glioblastoma? Understanding the Nature of Biological and Statistical Variability in Glioblastoma Expression Data. PloS one 6: e20826. 21829433 10.1371/journal.pone.0020826 PMC3145641

