# Supplementary figures and images for: G-Cimp Status Prediction Of Glioblastoma Samples Using mRNA Expression Data
Source: PLoS One. 2012 Nov 6;7(11):e47839. doi: 10.1371/journal.pone.0047839 (PMC3490960; doi:10.1371/journal.pone.0047839)

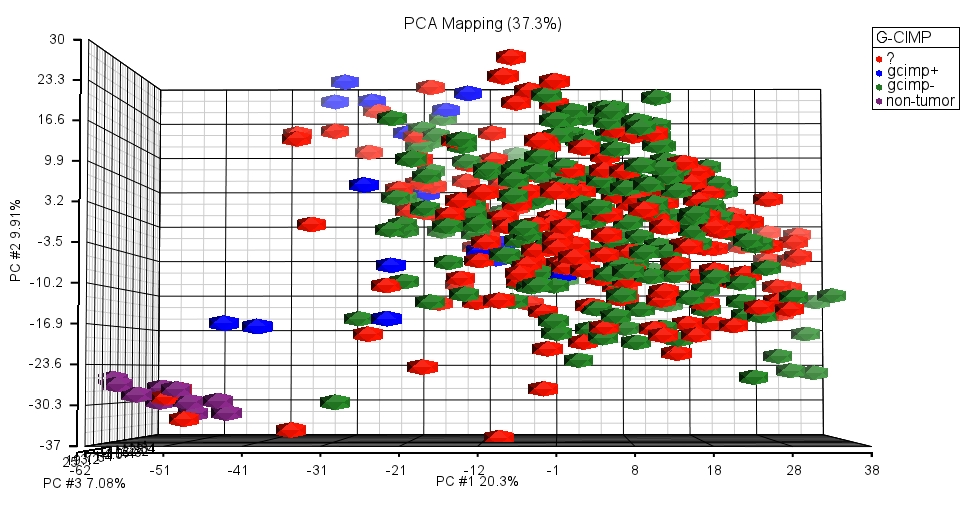


Figure S1

Supplement: Figure S1 — 3 TCGA GBM samples are clustered with normal brain samples and filtered out from TCGA expression data set. (DOC) [file pone.0047839.s001.doc]

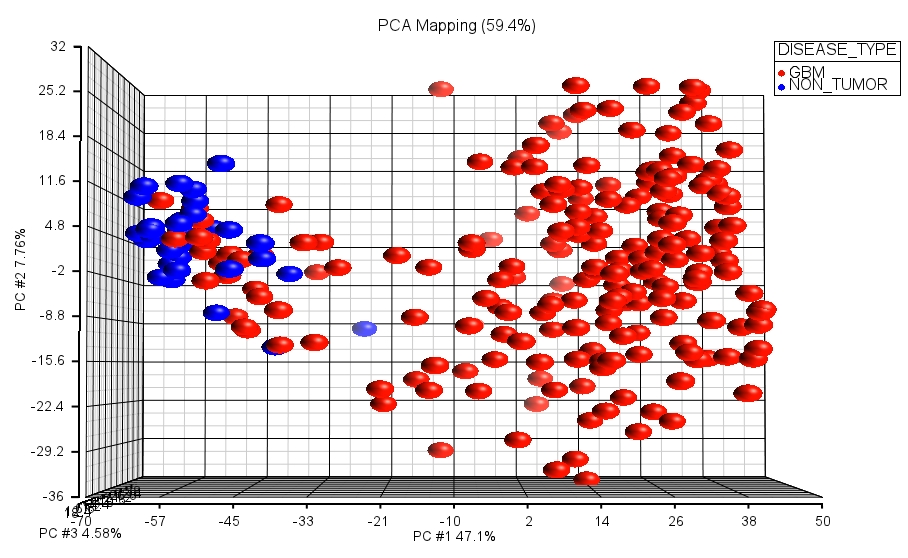


Figure S2

Supplement: Figure S2 — 27 NOB GBM samples are clustered with normal brain samples and filtered out from NOB expression data set. (DOC) [file pone.0047839.s002.doc]

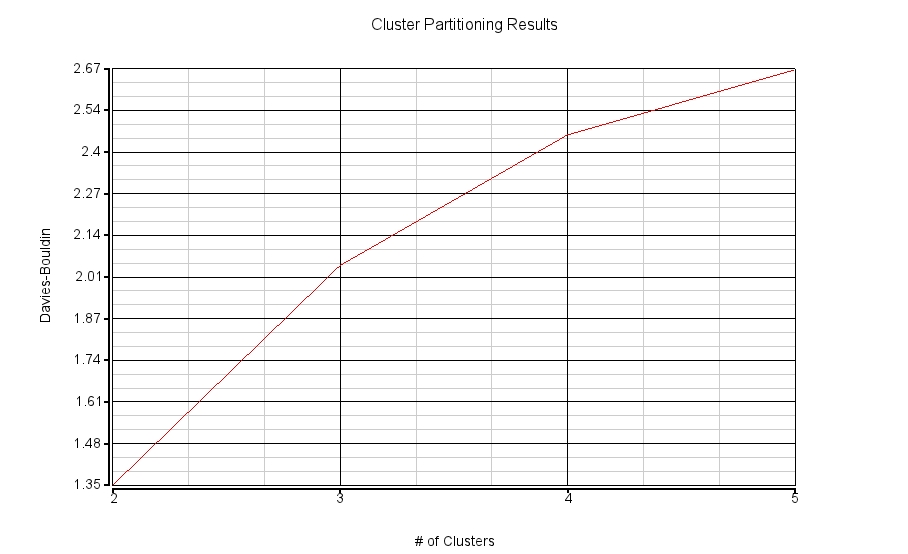


Figure S3

Supplement: Figure S3 — Davies-Boudin clustering performance score for clustering on TCGA methylation data for various number of clusters. Clustering separation is better when this score is lower. (DOC) [file pone.0047839.s003.doc]

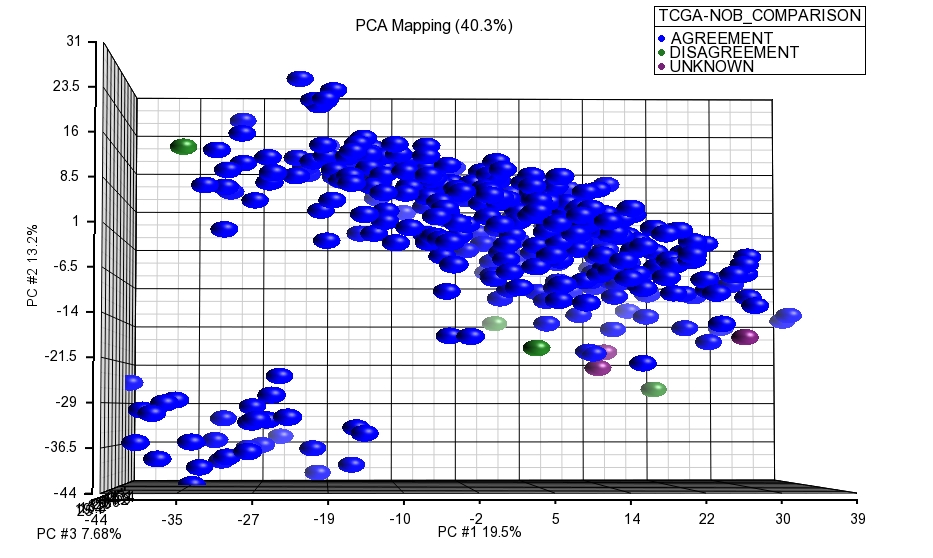


Figure S4

Supplement: Figure S4 — 1438 methylation sites. Four samples show disagreement between TCGA and our G-CIMP calls. (DOC) [file pone.0047839.s004.doc]

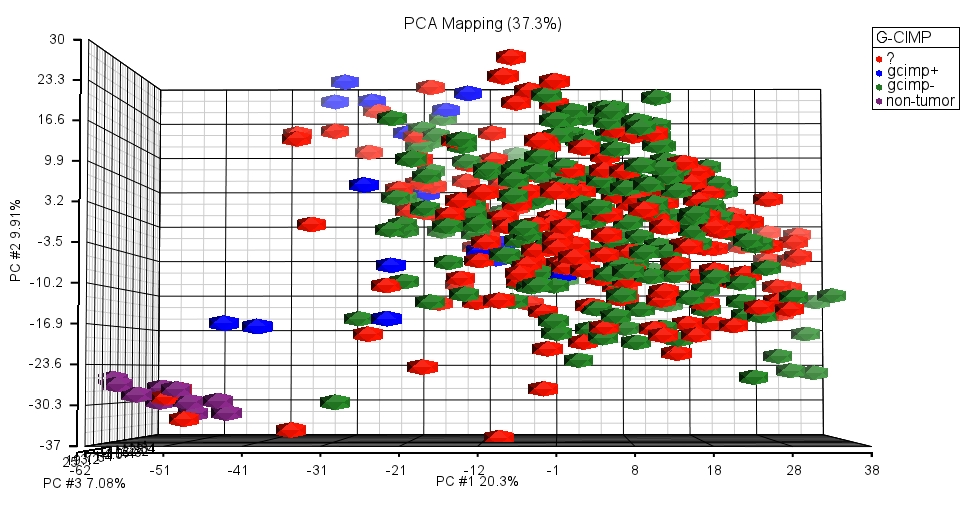


Figure S5

Supplement: Figure S5 — PCA plot of TCGA GBM and normal brain samples based on gene expression data. (DOC) [file pone.0047839.s005.doc]

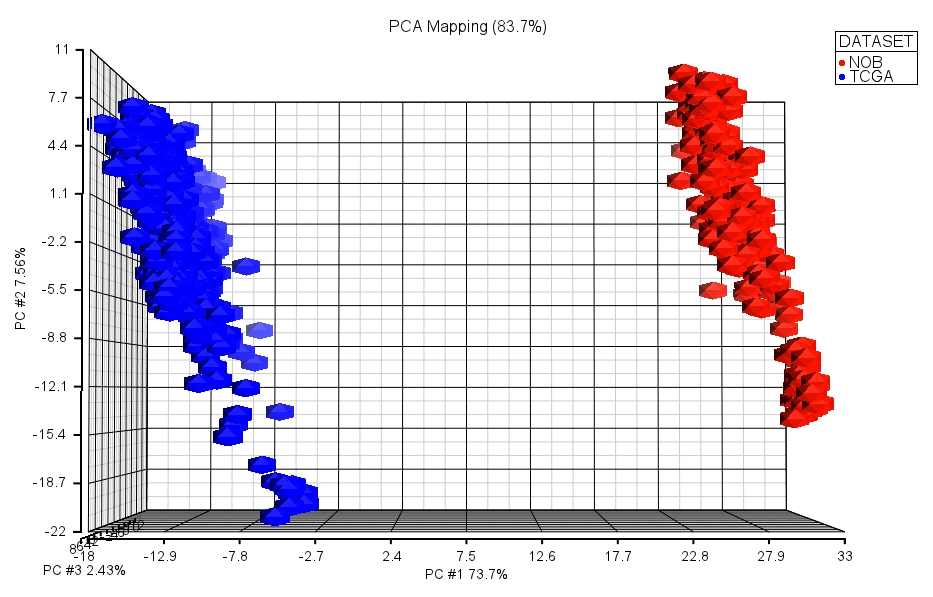


Figure S6-A


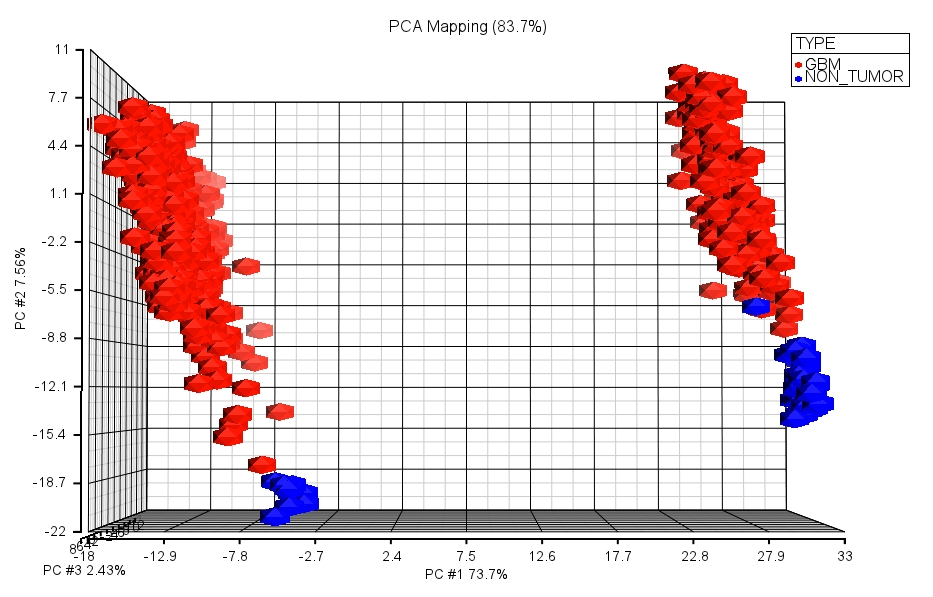


Figure S6-B


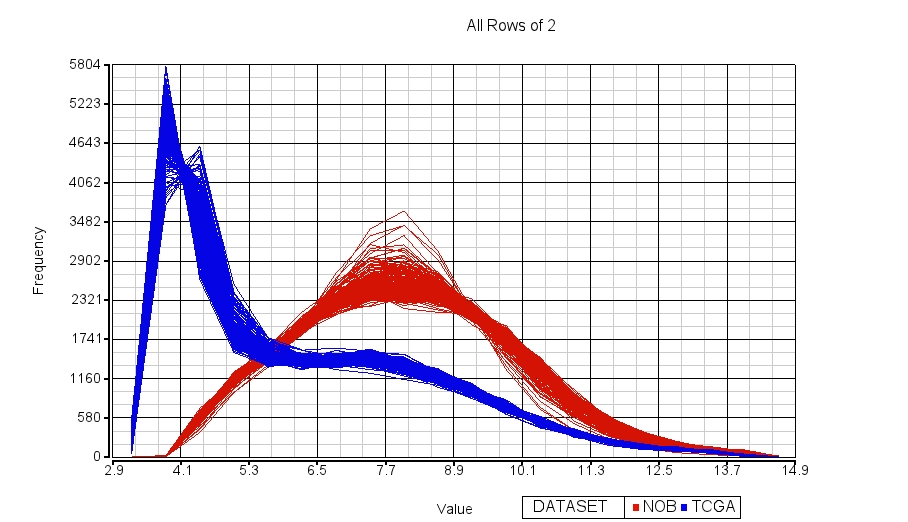


Figure S6-C

Supplement: Figure S6 — (A,B) PCA plots of GBM and normal brain samples in TCGA and NOB gene expression data sets before batch effect removal. 506 probe sets with largest variation are shown (C) Sample histogram of GBM and normal brain samples in TCGA and NOB gene expression data sets. We have used common 22,277 probe sets between Affymetrix U133 Plus 2.0 and Affymetrix U133A platforms. (DOC) [file pone.0047839.s006.doc]

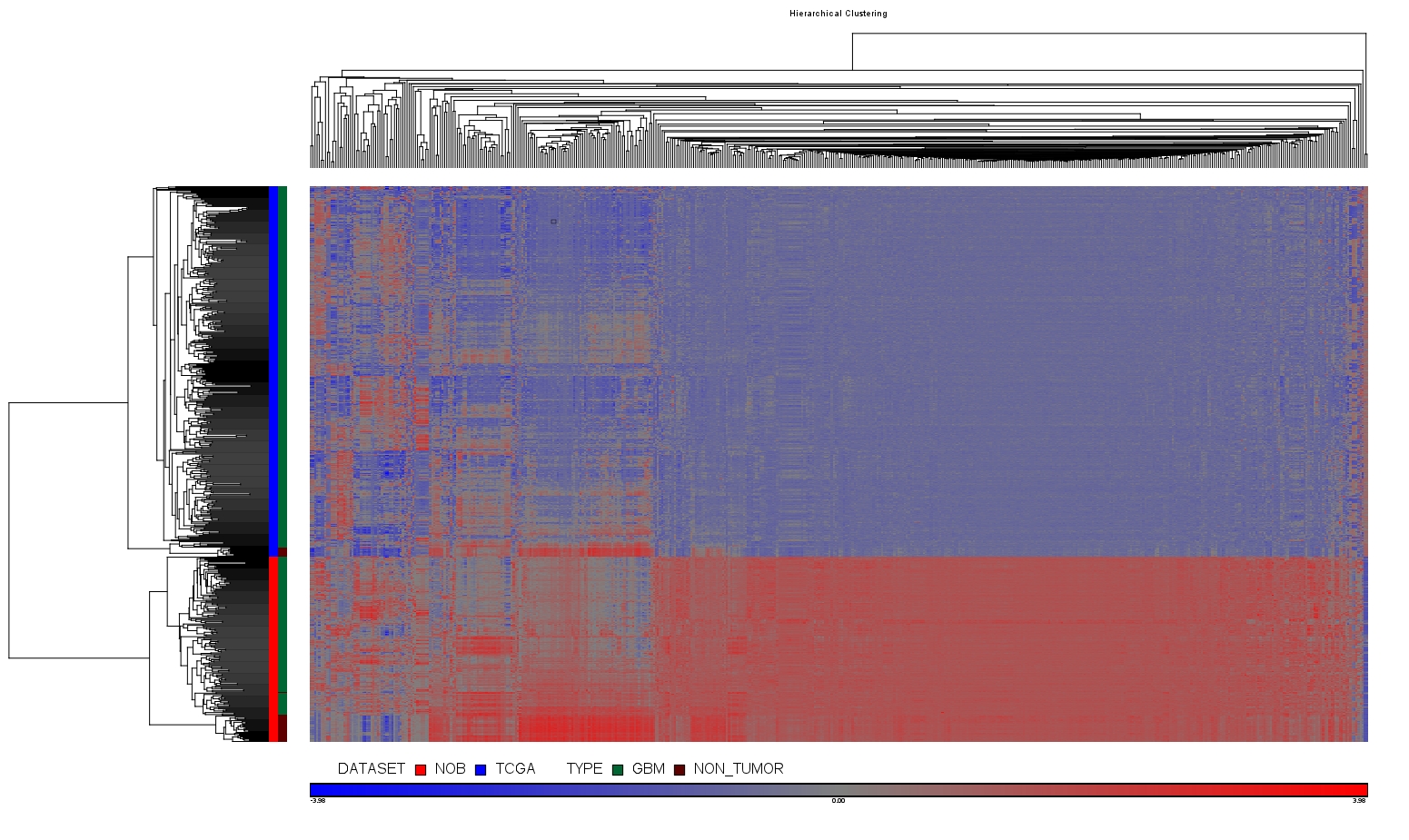


Figure S7

Supplement: Figure S7 — Hierarchical clustering of GBM and normal brain samples in TCGA and NOB gene expression data sets before batch effect removal. We used high variation 506 probe sets of combined data set for hierarchical clustering. (DOC) [file pone.0047839.s007.doc]

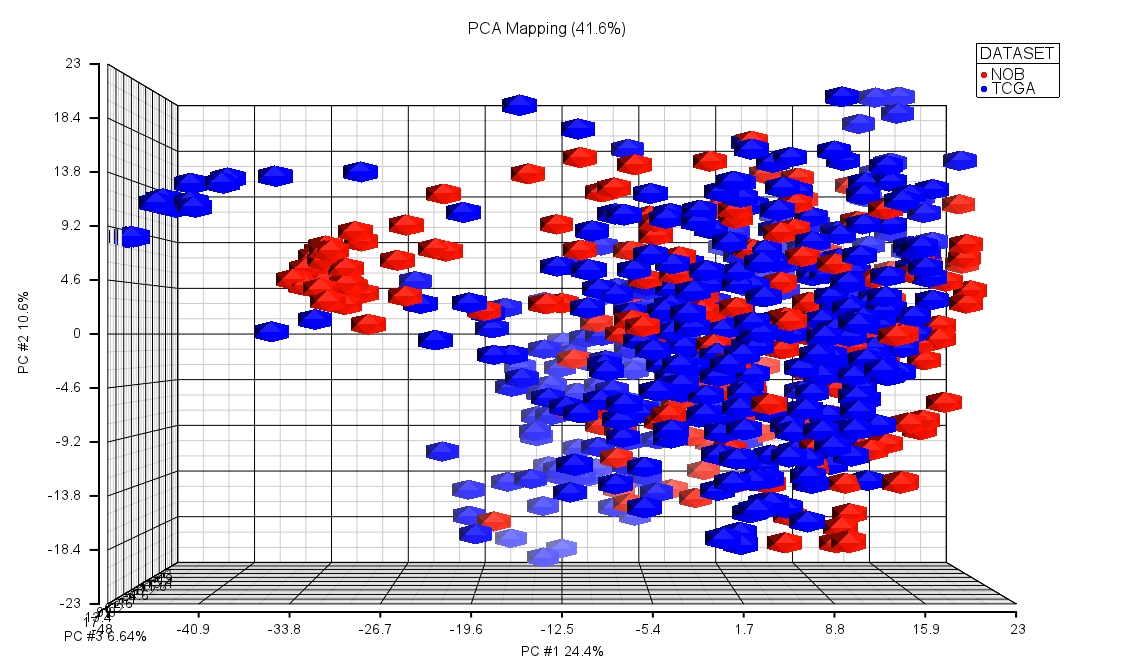


Figure S8-A


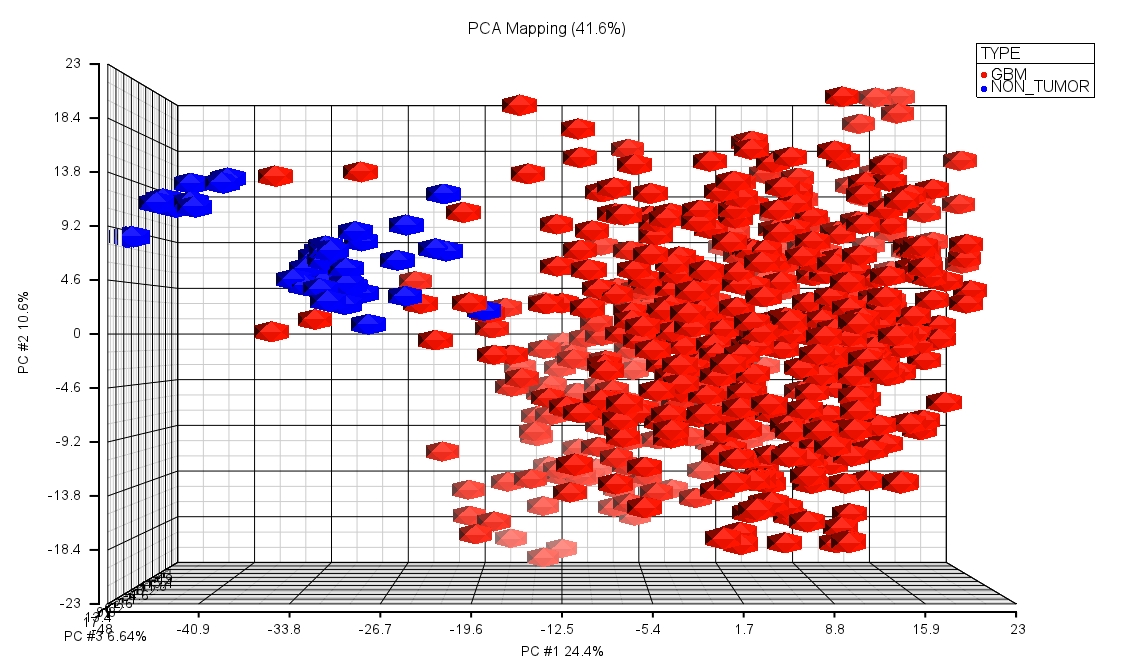


Figure S8-B


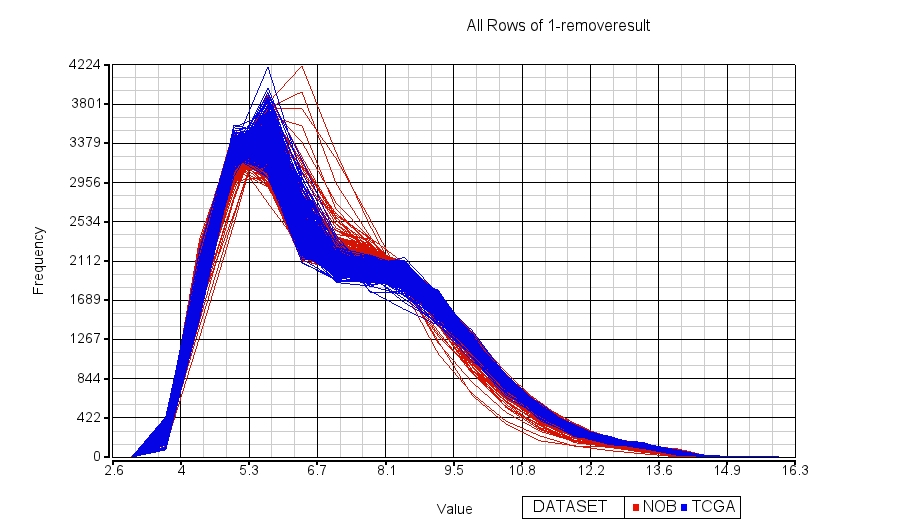


Figure S8-C

Supplement: Figure S8 — (A,B) PCA plots of GBM and normal brain samples in TCGA and NOB gene expression data sets after batch effect removal. 742 probe sets with largest variation are shown (C) Sample histogram of GBM and normal brain samples in TCGA and NOB gene expression data sets. We have used common 22,277 probe sets between Affymetrix U133 Plus 2.0 and Affymetrix U133A platforms. (DOC) [file pone.0047839.s008.doc]

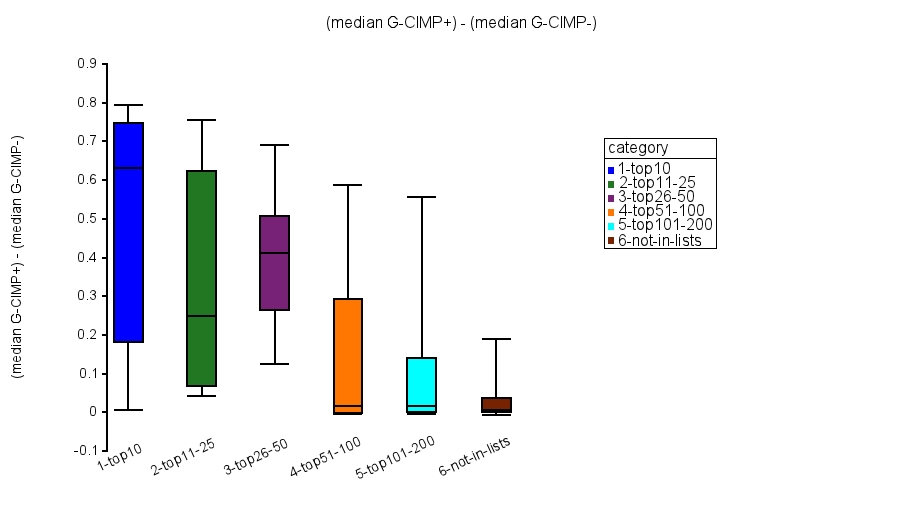


Figure S9

Supplement: Figure S9 — Methylation sites in different categories are represented with respect to absolute median methylation difference between G-CIMP positive and G-CIMP negative subtypes. (DOC) [file pone.0047839.s009.doc]

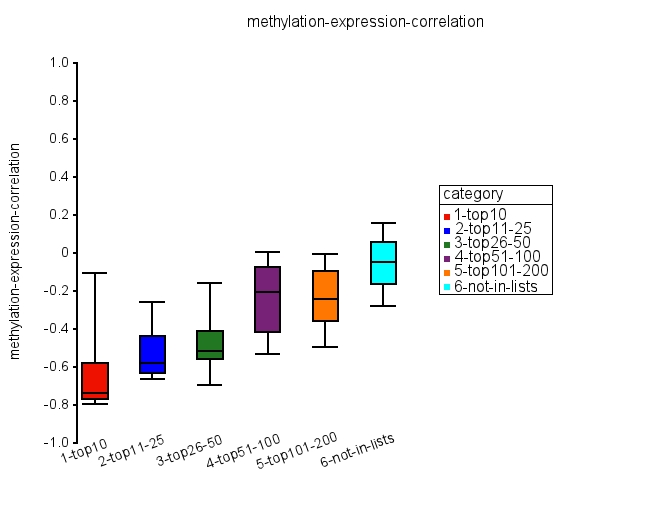


Figure S10

Supplement: Figure S10 — Methylation sites in different categories are represented with respect to methylation-expression correlations (Pearson). (DOC) [file pone.0047839.s010.doc]

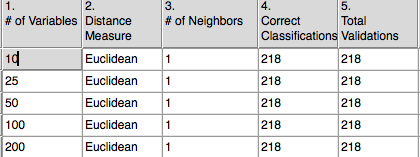


**Table S6**

Supplement: Table S6 — Prediction accuracy of the G-CIMP prediction method based on two-fold cross validation on the samples with known G-CIMP calls. (DOCX) [file pone.0047839.s016.docx]
