# Supplementary material for: G-Cimp Status Prediction Of Glioblastoma Samples Using mRNA Expression Data
Source: PLoS One. 2012 Nov 6;7(11):e47839. doi: 10.1371/journal.pone.0047839 (PMC3490960; doi:10.1371/journal.pone.0047839)
Supplement: Table S1 — Prediction models with 10 probe sets. (DOCX) [file pone.0047839.s011.docx]

Gene Symbol Selected Variable Fold-Change(gcimp+ vs. gcimp-) Gene Symbol

MSN 200600_at -3.84837 MSN

TAGLN2 200916_at -4.85077 TAGLN2

TIMP1 201666_at -7.70662 TIMP1

DYNLT3 203303_at -4.68229 DYNLT3

RBP1 203423_at -19.8095 RBP1

EMP3 203729_at -10.8023 EMP3

TRIP4 203732_at -3.97146 TRIP4

EFEMP2 206580_s_at -7.91895 EFEMP2

LGALS8 208933_s_at -4.56174 LGALS8

LGALS8 208935_s_at -2.82979 LGALS8
