# Supplementary material for: G-Cimp Status Prediction Of Glioblastoma Samples Using mRNA Expression Data
Source: PLoS One. 2012 Nov 6;7(11):e47839. doi: 10.1371/journal.pone.0047839 (PMC3490960; doi:10.1371/journal.pone.0047839)
Supplement: Table S2 — Prediction models with 25 probe sets. (DOCX) [file pone.0047839.s012.docx]

Gene Symbol Selected Variable Fold-Change(gcimp+ vs. gcimp-) Gene Symbol

MSN 200600_at -3.84837 MSN

TAGLN2 200916_at -4.85077 TAGLN2

DCTD 201572_x_at -2.5342 DCTD

TIMP1 201666_at -7.70662 TIMP1

DYNLT3 203303_at -4.68229 DYNLT3

RBP1 203423_at -19.8095 RBP1

EMP3 203729_at -10.8023 EMP3

TRIP4 203732_at -3.97146 TRIP4

TOM1L1 204485_s_at -5.53784 TOM1L1

EFEMP2 206580_s_at -7.91895 EFEMP2

CLIC1 208659_at -3.68923 CLIC1

LGALS8 208933_s_at -4.56174 LGALS8

LGALS8 208935_s_at -2.82979 LGALS8

LGALS3 208949_s_at -7.4266 LGALS3

CBR1 209213_at -5.5317 CBR1

EFEMP2 209356_x_at -6.57018 EFEMP2

DCTD 210137_s_at -2.58522 DCTD

FKBP9 212169_at -3.77166 FKBP9

SLC43A3 213113_s_at -3.65345 SLC43A3

KIAA0495 213340_s_at -6.40741 KIAA0495

LOC390940 213556_at -5.43223 LOC390940

MT1M 217546_at -12.5124 MT1M

FBXO17 /// SARS2 220233_at -3.52513 FBXO17 /// SARS2

SLC2A10 221024_s_at -5.49568 SLC2A10

PDPN 221898_at -13.0604 PDPN
