# Supplementary material for: G-Cimp Status Prediction Of Glioblastoma Samples Using mRNA Expression Data
Source: PLoS One. 2012 Nov 6;7(11):e47839. doi: 10.1371/journal.pone.0047839 (PMC3490960; doi:10.1371/journal.pone.0047839)
Supplement: Table S3 — Prediction models with 50 probe sets. (DOCX) [file pone.0047839.s013.docx]

Gene Symbol Selected Variable Fold-Change(gcimp+ vs. gcimp-) Gene Symbol

MSN 200600_at -3.84837 MSN

LDHA 200650_s_at -2.74049 LDHA

TAGLN2 200916_at -4.85077 TAGLN2

LGALS1 201105_at -3.25973 LGALS1

MYL12A 201319_at -2.47894 MYL12A

DCTD 201572_x_at -2.5342 DCTD

TIMP1 201666_at -7.70662 TIMP1

FABP5 202345_s_at -11.8057 FABP5

IGFBP2 202718_at -8.02305 IGFBP2

DYNLT3 203303_at -4.68229 DYNLT3

RBP1 203423_at -19.8095 RBP1

PGCP 203501_at -3.43814 PGCP

EMP3 203729_at -10.8023 EMP3

TRIP4 203732_at -3.97146 TRIP4

MAOB 204041_at -8.02068 MAOB

SLC25A24 204342_at -3.20484 SLC25A24

TOM1L1 204485_s_at -5.53784 TOM1L1

PDPN 204879_at -9.08682 PDPN

AKAP6 205359_at 2.28413 AKAP6

EFEMP2 206580_s_at -7.91895 EFEMP2

CLIC1 208659_at -3.68923 CLIC1

LGALS8 208933_s_at -4.56174 LGALS8

LGALS8 208935_s_at -2.82979 LGALS8

LGALS8 208936_x_at -2.61364 LGALS8

LGALS3 208949_s_at -7.4266 LGALS3

MYD88 209124_at -2.18319 MYD88

CBR1 209213_at -5.5317 CBR1

SWAP70 209306_s_at -2.73384 SWAP70

EFEMP2 209356_x_at -6.57018 EFEMP2

CHI3L1 209395_at -19.1248 CHI3L1

CHI3L1 209396_s_at -22.7002 CHI3L1

CYP2E1 209975_at 2.16294 CYP2E1

DCTD 210137_s_at -2.58522 DCTD

TAGLN2 210978_s_at -3.70858 TAGLN2

FKBP9 212169_at -3.77166 FKBP9

MT1E 212859_x_at -4.1286 MT1E

SLC43A3 213113_s_at -3.65345 SLC43A3

KIAA0495 213340_s_at -6.40741 KIAA0495

CDHR1 213369_at 1.89738 CDHR1

LOC390940 213556_at -5.43223 LOC390940

MT1M 217546_at -12.5124 MT1M

TMBIM1 217730_at -2.95314 TMBIM1

XKR8 218753_at -1.91935 XKR8

TMEM22 219569_s_at -4.1328 TMEM22

FBXO17 /// SARS2 220233_at -3.52513 FBXO17 /// SARS2

GREB1L 220340_at 1.79051 GREB1L

SLC2A10 221024_s_at -5.49568 SLC2A10

MOSC2 221636_s_at -3.16903 MOSC2

PDPN 221898_at -13.0604 PDPN

C19orf66 53720_at -3.10597 C19orf66
