# Supplementary material for: G-Cimp Status Prediction Of Glioblastoma Samples Using mRNA Expression Data
Source: PLoS One. 2012 Nov 6;7(11):e47839. doi: 10.1371/journal.pone.0047839 (PMC3490960; doi:10.1371/journal.pone.0047839)
Supplement: Table S4 — Prediction models with 100 probe sets. (DOCX) [file pone.0047839.s014.docx]

Gene Symbol Selected Variable Fold-Change(gcimp+ vs. gcimp-) Gene Symbol

MSN 200600_at -3.84837 MSN

LDHA 200650_s_at -2.74049 LDHA

ANXA5 200782_at -1.99617 ANXA5

TAGLN2 200916_at -4.85077 TAGLN2

LGALS1 201105_at -3.25973 LGALS1

PLP2 201136_at -5.07121 PLP2

PLS3 201215_at -3.03499 PLS3

MYL12A 201319_at -2.47894 MYL12A

DCTD 201571_s_at -3.03978 DCTD

DCTD 201572_x_at -2.5342 DCTD

ANXA2 201590_x_at -3.53262 ANXA2

TIMP1 201666_at -7.70662 TIMP1

FABP5 202345_s_at -11.8057 FABP5

IGFBP2 202718_at -8.02305 IGFBP2

DYNLT3 203303_at -4.68229 DYNLT3

PRPS2 203401_at -2.80826 PRPS2

RBP1 203423_at -19.8095 RBP1

PGCP 203501_at -3.43814 PGCP

SLC25A20 203658_at -2.72206 SLC25A20

EMP3 203729_at -10.8023 EMP3

TRIP4 203732_at -3.97146 TRIP4

MAOB 204041_at -8.02068 MAOB

SLC25A24 204342_at -3.20484 SLC25A24

F3 204363_at -4.08487 F3

TOM1L1 204485_s_at -5.53784 TOM1L1

PDPN 204879_at -9.08682 PDPN

CD58 205173_x_at -3.26442 CD58

AKAP6 205359_at 2.28413 AKAP6

CBLN1 205747_at 1.59158 CBLN1

TNFAIP6 206026_s_at -5.99959 TNFAIP6

EFEMP2 206580_s_at -7.91895 EFEMP2

PGCP 208454_s_at -2.64642 PGCP

CLIC1 208659_at -3.68923 CLIC1

PTRF 208789_at -3.2554 PTRF

ANXA2P2 208816_x_at -3.38834 ANXA2P2

LGALS8 208933_s_at -4.56174 LGALS8

LGALS8 208934_s_at -2.85734 LGALS8

LGALS8 208935_s_at -2.82979 LGALS8

LGALS8 208936_x_at -2.61364 LGALS8

SEPHS1 208939_at 2.15519 SEPHS1

LGALS3 208949_s_at -7.4266 LGALS3

ABI1 209028_s_at 2.00217 ABI1

MDK 209035_at -3.79551 MDK

MYD88 209124_at -2.18319 MYD88

TRIP6 209129_at -3.48128 TRIP6

CBR1 209213_at -5.5317 CBR1

SWAP70 209306_s_at -2.73384 SWAP70

EFEMP2 209356_x_at -6.57018 EFEMP2

CHI3L1 209395_at -19.1248 CHI3L1

CHI3L1 209396_s_at -22.7002 CHI3L1

RARRES2 209496_at -7.89077 RARRES2

CYP2E1 209975_at 2.16294 CYP2E1

PARD3 210094_s_at 2.10241 PARD3

DCTD 210137_s_at -2.58522 DCTD

PMP22 210139_s_at -2.85295 PMP22

ANXA2 210427_x_at -3.59038 ANXA2

TAGLN2 210978_s_at -3.70858 TAGLN2

SERPINB6 211474_s_at -2.07495 SERPINB6

FKBP9 212169_at -3.77166 FKBP9

GNG12 212294_at -2.35511 GNG12

KHNYN 212355_at -2.78718 KHNYN

MT1E 212859_x_at -4.1286 MT1E

SLC43A3 213113_s_at -3.65345 SLC43A3

ZNF248 213269_at 2.65109 ZNF248

KIAA0495 213340_s_at -6.40741 KIAA0495

CDHR1 213369_at 1.89738 CDHR1

ANXA2 213503_x_at -3.59068 ANXA2

LOC390940 213556_at -5.43223 LOC390940

YIPF1 214733_s_at -1.89115 YIPF1

--- 215180_at 2.36333 ---

ZNF804A 215767_at 3.92916 ZNF804A

CBARA1 216903_s_at 2.06893 CBARA1

MT1M 217546_at -12.5124 MT1M

TMBIM1 217730_at -2.95314 TMBIM1

WAC 217742_s_at 1.81605 WAC

PPCS 218341_at -2.07383 PPCS

C19orf66 218429_s_at -2.38905 C19orf66

H2AFY2 218445_at 2.60108 H2AFY2

HEBP1 218450_at -2.74929 HEBP1

ECHDC2 218552_at -3.70614 ECHDC2

XKR8 218753_at -1.91935 XKR8

FERMT1 218796_at 7.68534 FERMT1

CCDC109B 218802_at -4.09234 CCDC109B

MTPAP 218947_s_at 2.20453 MTPAP

C13orf18 219471_at -4.06491 C13orf18

TMEM22 219569_s_at -4.1328 TMEM22

RANBP17 219661_at 1.46527 RANBP17

FBXO17 /// SARS2 220233_at -3.52513 FBXO17 /// SARS2

GREB1L 220340_at 1.79051 GREB1L

SLC2A10 221024_s_at -5.49568 SLC2A10

PARD3 221526_x_at 2.05885 PARD3

PARD3 221527_s_at 2.7123 PARD3

MOSC2 221636_s_at -3.16903 MOSC2

JMJD1C 221763_at 2.22784 JMJD1C

MARCH8 221824_s_at 2.54315 MARCH8

PDPN 221898_at -13.0604 PDPN

NSUN6 222128_at 2.14228 NSUN6

C13orf18 44790_s_at -3.54614 C13orf18

C19orf66 53720_at -3.10597 C19orf66

FERMT1 60474_at 7.64535 FERMT1
