# Supplementary material for: G-Cimp Status Prediction Of Glioblastoma Samples Using mRNA Expression Data
Source: PLoS One. 2012 Nov 6;7(11):e47839. doi: 10.1371/journal.pone.0047839 (PMC3490960; doi:10.1371/journal.pone.0047839)
Supplement: Table S5 — Prediction models with 200 probe sets. (DOCX) [file pone.0047839.s015.docx]

Gene Symbol Selected Variable Fold-Change(gcimp+ vs. gcimp-) Gene Symbol

CYP2E1 1431_at 1.28093 CYP2E1

MSN 200600_at -3.84837 MSN

CSRP1 200621_at -2.92466 CSRP1

LDHA 200650_s_at -2.74049 LDHA

LITAF 200704_at -2.20606 LITAF

LITAF 200706_s_at -2.12654 LITAF

ANXA5 200782_at -1.99617 ANXA5

TAGLN2 200916_at -4.85077 TAGLN2

ANXA1 201012_at -4.54428 ANXA1

LGALS1 201105_at -3.25973 LGALS1

PLP2 201136_at -5.07121 PLP2

BHLHE40 201170_s_at -3.18687 BHLHE40

PLS3 201215_at -3.03499 PLS3

MYL12A 201319_at -2.47894 MYL12A

DCTD 201571_s_at -3.03978 DCTD

DCTD 201572_x_at -2.5342 DCTD

ANXA2 201590_x_at -3.53262 ANXA2

TIMP1 201666_at -7.70662 TIMP1

AEBP1 201792_at -4.76775 AEBP1

NET1 201829_at 2.45587 NET1

NET1 201830_s_at 3.28334 NET1

CAPG 201850_at -2.86878 CAPG

PLAT 201860_s_at -3.92102 PLAT

UBXN1 201871_s_at 1.50338 UBXN1

ACAA2 202003_s_at -2.25279 ACAA2

WWTR1 202133_at -3.02927 WWTR1

ZMYND11 202136_at 1.72937 ZMYND11

FABP5 202345_s_at -11.8057 FABP5

S100A13 202598_at -4.05901 S100A13

IGFBP2 202718_at -8.02305 IGFBP2

ADM 202912_at -6.48146 ADM

UPP1 203234_at -3.61761 UPP1

DRG2 203267_s_at -1.85181 DRG2

DYNLT3 203303_at -4.68229 DYNLT3

PRPS2 203401_at -2.80826 PRPS2

RBP1 203423_at -19.8095 RBP1

LDB1 203451_at 1.63303 LDB1

PGCP 203501_at -3.43814 PGCP

UPF2 203519_s_at 2.1314 UPF2

FCHSD2 203620_s_at 1.96012 FCHSD2

SLC25A20 203658_at -2.72206 SLC25A20

EMP3 203729_at -10.8023 EMP3

TRIP4 203732_at -3.97146 TRIP4

CYP27A1 203979_at -1.90569 CYP27A1

MAOB 204041_at -8.02068 MAOB

SLC25A24 204342_at -3.20484 SLC25A24

AK3L1 204348_s_at -3.08388 AK3L1

F3 204363_at -4.08487 F3

TOM1L1 204485_s_at -5.53784 TOM1L1

DPYD 204646_at -4.10032 DPYD

PHF16 204866_at 2.44157 PHF16

PDPN 204879_at -9.08682 PDPN

ICAM3 204949_at -2.84386 ICAM3

PARG 205060_at 1.45343 PARG

CD58 205173_x_at -3.26442 CD58

BMP2 205289_at 3.26113 BMP2

BMP2 205290_s_at 4.16029 BMP2

AKAP6 205359_at 2.28413 AKAP6

PDGFA 205463_s_at -4.10228 PDGFA

CBLN1 205747_at 1.59158 CBLN1

CPEB3 205773_at 1.56233 CPEB3

TNFAIP6 206025_s_at -5.23202 TNFAIP6

TNFAIP6 206026_s_at -5.99959 TNFAIP6

MEOX2 206201_s_at -9.9235 MEOX2

IDS 206342_x_at -2.53673 IDS

EFEMP2 206580_s_at -7.91895 EFEMP2

KLRC1 /// KLRC2 206785_s_at 15.2618 KLRC1 /// KLRC2

HMX1 207353_s_at 1.58077 HMX1

NR2E1 207443_at -6.12013 NR2E1

PGCP 208454_s_at -2.64642 PGCP

CLIC1 208659_at -3.68923 CLIC1

TFRC 208691_at -2.34968 TFRC

FSTL1 208782_at -2.43391 FSTL1

PTRF 208789_at -3.2554 PTRF

ANXA2P2 208816_x_at -3.38834 ANXA2P2

H1F0 208886_at 2.27523 H1F0

LGALS8 208933_s_at -4.56174 LGALS8

LGALS8 208934_s_at -2.85734 LGALS8

LGALS8 208935_s_at -2.82979 LGALS8

LGALS8 208936_x_at -2.61364 LGALS8

SEPHS1 208939_at 2.15519 SEPHS1

SEPHS1 208940_at 2.26801 SEPHS1

LGALS3 208949_s_at -7.4266 LGALS3

ABI1 209028_s_at 2.00217 ABI1

MDK 209035_at -3.79551 MDK

MYD88 209124_at -2.18319 MYD88

TRIP6 209129_at -3.48128 TRIP6

CBR1 209213_at -5.5317 CBR1

SWAP70 209306_s_at -2.73384 SWAP70

SWAP70 209307_at -2.50108 SWAP70

EFEMP2 209356_x_at -6.57018 EFEMP2

FAM190B 209379_s_at 1.84818 FAM190B

CHI3L1 209395_at -19.1248 CHI3L1

CHI3L1 209396_s_at -22.7002 CHI3L1

GRB10 209409_at -3.16918 GRB10

RARRES2 209496_at -7.89077 RARRES2

RIPK2 209544_at 1.54001 RIPK2

PLA2G16 209581_at -2.60447 PLA2G16

PNPLA4 209739_s_at -3.68283 PNPLA4

SEC31B 209889_at 1.88263 SEC31B

CYP2E1 209975_at 2.16294 CYP2E1

PARD3 210094_s_at 2.10241 PARD3

DCTD 210137_s_at -2.58522 DCTD

PMP22 210139_s_at -2.85295 PMP22

ANXA2 210427_x_at -3.59038 ANXA2

--- 210524_x_at -2.55667 ---

SLC43A3 210692_s_at -2.03553 SLC43A3

RGN 210751_s_at -3.39434 RGN

CDH20 210913_at 1.63121 CDH20

TAGLN2 210978_s_at -3.70858 TAGLN2

KCNB1 211006_s_at 3.67145 KCNB1

WDR37 211383_s_at 1.79843 WDR37

SERPINB6 211474_s_at -2.07495 SERPINB6

PDLIM4 211564_s_at -7.77345 PDLIM4

BDH1 211715_s_at -2.1009 BDH1

CD58 211744_s_at -3.00187 CD58

MYST4 211874_s_at 2.03535 MYST4

FKBP9 212169_at -3.77166 FKBP9

IFITM3 212203_x_at -2.69401 IFITM3

GNG12 212294_at -2.35511 GNG12

SEL1L3 212314_at -4.64806 SEL1L3

KHNYN 212355_at -2.78718 KHNYN

KHNYN 212356_at -2.08799 KHNYN

KIAA1279 212453_at 1.76039 KIAA1279

MYST4 212462_at 2.02703 MYST4

ZNF609 212618_at 1.51308 ZNF609

MT1E 212859_x_at -4.1286 MT1E

SLC43A3 213113_s_at -3.65345 SLC43A3

PION 213142_x_at -3.22679 PION

ZNF248 213269_at 2.65109 ZNF248

KIAA0495 213340_s_at -6.40741 KIAA0495

CDHR1 213369_at 1.89738 CDHR1

ARL3 213433_at 1.56124 ARL3

ANXA2 213503_x_at -3.59068 ANXA2

LOC390940 213556_at -5.43223 LOC390940

MT1F 213629_x_at -2.89341 MT1F

LOC284244 214162_at 2.22842 LOC284244

PDLIM4 214175_x_at -6.83504 PDLIM4

MYST4 214496_x_at 2.01749 MYST4

YIPF1 214733_s_at -1.89115 YIPF1

NMT2 215069_at 1.46791 NMT2

--- 215180_at 2.36333 ---

--- 215473_at 1.66649 ---

ZNF492 215532_x_at 1.42094 ZNF492

NMT2 215743_at 1.48445 NMT2

ZNF804A 215767_at 3.92916 ZNF804A

PLA2G5 215870_s_at -8.28278 PLA2G5

--- 216093_at 1.71417 ---

CBARA1 216903_s_at 2.06893 CBARA1

CD58 216942_s_at -2.81484 CD58

MT1M 217546_at -12.5124 MT1M

TMBIM1 217730_at -2.95314 TMBIM1

WAC 217742_s_at 1.81605 WAC

BCCIP 218264_at 1.74824 BCCIP

C10orf18 218331_s_at 1.8909 C10orf18

PPCS 218341_at -2.07383 PPCS

STEAP3 218424_s_at -3.46341 STEAP3

C19orf66 218429_s_at -2.38905 C19orf66

H2AFY2 218445_at 2.60108 H2AFY2

HEBP1 218450_at -2.74929 HEBP1

SLC2A4RG 218494_s_at -2.17987 SLC2A4RG

ECHDC2 218552_at -3.70614 ECHDC2

C10orf2 218590_at 1.81398 C10orf2

XKR8 218753_at -1.91935 XKR8

FERMT1 218796_at 7.68534 FERMT1

CCDC109B 218802_at -4.09234 CCDC109B

SIRT1 218878_s_at 1.93471 SIRT1

MTPAP 218947_s_at 2.20453 MTPAP

CUTC 218970_s_at 1.82196 CUTC

SEMA4G 219194_at 1.48504 SEMA4G

KLHL26 219354_at -2.5179 KLHL26

MSRB2 219451_at 2.38483 MSRB2

C13orf18 219471_at -4.06491 C13orf18

COX15 219547_at 1.768 COX15

TMEM22 219569_s_at -4.1328 TMEM22

ACSS3 219616_at -4.68298 ACSS3

RANBP17 219661_at 1.46527 RANBP17

WAC 219679_s_at 2.01226 WAC

ZC4H2 220040_x_at 2.3044 ZC4H2

FBXO17 /// SARS2 220233_at -3.52513 FBXO17 /// SARS2

GREB1L 220340_at 1.79051 GREB1L

FAR2 220615_s_at -2.35485 FAR2

SLC2A10 221024_s_at -5.49568 SLC2A10

PARD3 221526_x_at 2.05885 PARD3

PARD3 221527_s_at 2.7123 PARD3

BCAN 221623_at 3.66589 BCAN

MOSC2 221636_s_at -3.16903 MOSC2

JMJD1C 221763_at 2.22784 JMJD1C

PHF11 221816_s_at -2.74783 PHF11

MARCH8 221824_s_at 2.54315 MARCH8

PDPN 221898_at -13.0604 PDPN

NSUN6 222128_at 2.14228 NSUN6

PION 222150_s_at -3.28555 PION

SLC27A3 222217_s_at -2.97592 SLC27A3

LOC282997 222307_at 1.52546 LOC282997

LDB1 35160_at 1.63972 LDB1

C13orf18 44790_s_at -3.54614 C13orf18

SSH3 51192_at -1.70758 SSH3

C19orf66 53720_at -3.10597 C19orf66

FERMT1 60474_at 7.64535 FERMT1
